# The Influence of COVID on Emergency Medicine Career Choice: A Survey of Medical Students

**DOI:** 10.7759/cureus.59055

**Published:** 2024-04-26

**Authors:** Shruti Chandra, Mark Olaf, Megan Fix, Sharon Bord, Linda Katirji, William Dixon, Michael Pasirstein, Caitlin Schrepel, Kevin R Scott

**Affiliations:** 1 Emergency Medicine, Thomas Jefferson University, Philadelphia, USA; 2 Emergency Medicine, Geisinger Commonwealth School of Medicine, Scranton, USA; 3 Emergency Medicine, University of Utah School of Medicine, Salt Lake City, USA; 4 Emergency, Johns Hopkins University School of Medicine, Baltimore, USA; 5 Emergency Medicine, University of Kentucky, Lexington, USA; 6 Emergency Medicine, Stanford University Hospital, Palo Alto, USA; 7 Emergency Medicine, Sidney Kimmel Medical College Thomas Jefferson University, Philadelphia, USA; 8 Emergency Medicine, University of Washington School of Medicine, Seattle, USA; 9 Emergency Medicine, University of Pennsylvania Health System, Philadelphia, USA

**Keywords:** emergency medicine, covid, covid 19, medical student advising, career choice, medical student education

## Abstract

Background

The COVID-19 pandemic has led to substantial changes in the delivery of healthcare and medical education. Little is known about how the pandemic has altered medical students' perceptions in regard to career choice.

Methods

The authors developed and implemented a multi-center survey that evaluated medical students' preferred career choice before and during the coronavirus pandemic, as well as the influence of pandemic-related factors on that choice. The survey was distributed to all levels of medical students (MS) at nine medical schools across the country from November 2020 to January 2021 and represented a convenience sample. Preferred career choice was assessed through the use of a Likert scale and additional factors affecting career choice were solicited. The degree of interest before and during the pandemic, as well as factors influencing the shift, were treated as ordinal variables and compared using chi-squared testing. Cohen's Kappa statistic was calculated to assess the degree of shifts of interest in Emergency Medicine among students. The study was deemed exempt by the Institutional Review Board at the host institution, Sidney Kimmel Medical College at Thomas Jefferson University, and all participating sites.

Results

A total of 1431 of 6710 (21.3%) eligible students completed the survey. The COVID pandemic was cited as a reason for a changed interest in specialty by 193 (13.5%) students. The most common reason for specialty change was the students’ clinical experience, followed by a desire to be on the front lines, and personal/family health concerns. There was a significant association between career change and degree of interest among students interested in emergency medicine (EM) as their future specialty before the COVID pandemic as well as during the COVID pandemic. Living with an immunocompromised individual had a significant association with a reduced interest in EM. There was a significant association between EM rotation completion and how interested students were in EM as their future specialty before the COVID pandemic and during the COVID pandemic. Among EM-interested students whose specialty interest was changed by the COVID pandemic, 34 (41.5%) became less favorable to EM, 28 (34.2%) stayed the same, and 20 (24.4%) students became more favorable to EM.

Conclusions

The impact of COVID-19 on medical students’ career choice is a complicated matter that involves both personal and professional factors. It appears that there is a trend towards less interest in the field of EM with multifactorial influences, some of which are related to the COVID-19 pandemic.

## Introduction

Multiple factors influence medical student career choices including demographics, medical school characteristics, students’ perceptions of specialty characteristics, student-held values, family influences, and their experiences during medical school, among others [1-3]. In addition, prior work has suggested that global events such as pandemics, may impact medical student education and career selection [3]. In the early 1990s, a study of medical students looked at the impact of the HIV/AIDS epidemic on student career choices and indicated that students were overall willing to care for HIV-positive patients, but both surgery and emergency medicine (EM) were perceived as specialties with high risk of exposure. Despite this, no significant differences were found in career, training, and practice location [4]. More recently, a study from Brazil surveyed over 10,000 medical students and explored the impact of the COVID-19 pandemic on medical students’ motivations to be part of the healthcare team. The study found that students were motivated by a sense of purpose or duty, altruism, a perception of good performance, and values of professionalism more than their interest in learning, which may have an impact on career choice, although this was not the focus of this particular study [5].

Some studies have evaluated the effect of the COVID-19 pandemic on medical student career choice. One US survey study performed early in the pandemic found that one-fifth of surveyed medical students believed that the COVID-19 pandemic would affect their choice of specialty [6]. A study from China showed that the pandemic was associated with both a 10% increase and a nearly 7% decrease in willingness to be a doctor and both an increase and decrease in willingness to major in respiratory illnesses as a career path [7].

We are now beginning to understand how the COVID-19 pandemic has impacted medical students and their educational opportunities, which may also impact career choice. Recent work has suggested that the COVID-19 pandemic has led to higher levels of anxiety in medical students and educators have argued that it is paramount for advisors and mentors to “safeguard (medical student’s) mental health and implement effective strategies to support their educational, physical, mental, and professional well-being” [8]. Another recent commentary explored the effects of the pandemic on EM student education and discussed the disruptions in clinical duties and patient experience posed by the pandemic that may also have an impact on student career choice [9]. A letter to the editor in England described the educational changes and specifically the disruptions to anatomy education during the pandemic being a potential factor influencing medical students' career choices and futures [10]. A blog post on MedPageToday entitled “Still want to be a doctor post COVID-19?” highlights that the pandemic may affect medical school choice based on students’ desires to be on the frontlines [11].

In addition to the influence of the COVID-19 pandemic on the choice to become a physician, and its influence upon broad specialty choice as a physician, we suspect to see an influence upon career choice within Emergency Medicine. Emergency Medicine is largely considered a "front line" specialty which exposes clinicians to particular risks of violence and illness. During the COVID-19 pandemic, there were concerns about resource allocation and increased risks of contracting illness among front line clinicians, which may highlight elements of job-associated risk in Emergency Medicine, which previously may not have been influential in making career choices among medical students. In addition, shifts in the workforce during peaks and nadirs of the pandemic may have highlighted risks of the specialty which had not previously been influential to students.

Given these disruptions in medical education during the COVID-19 pandemic and the suggested impacts on medical students, it stands to reason that this global event will have some impact on medical student career and specialty selection, and particularly within Emergency Medicine. However, there remains a paucity of literature to explain how COVID-19 is impacting students' career choices.

The aim of this study was to understand the degree of influence of the COVID-19 pandemic upon the choice of Emergency Medicine as a career choice among medical students at a sample of medical schools within the United States. In order to ascertain the influence of the COVID-19 pandemic on career choice, students' self-stated initial career interests were used as baseline measures. The authors conducted a multi-institutional survey of all levels of medical students to determine overall baseline career interest in all specialties before the pandemic and career interest during the pandemic. Specific survey questions focused on students’ affinity for a career in Emergency Medicine (EM), and factors contributing to interest in or away from EM as this was considered a specialty on the “frontlines” during the pandemic. Given the "frontline" nature of EM, it was hypothesized that shifts towards or away from EM might be substantial.

This article was previously posted to the Research Square preprint server on June 26, 2023.

## Materials and methods

Study aim and design

The authors sought to evaluate the impact of the COVID-19 pandemic upon career specialty choice among medical students in the United States. The study was a multi-center consensus-driven survey study with students from a convenience sample of nine medical schools across the United States. The study was considered Institutional Review Board exempted at all participating institutions (see author affiliations for school listings), and was granted exemption Control #20E.851 at the primary study institution, Thomas Jefferson University.

Survey instrument

**Figure 1:**
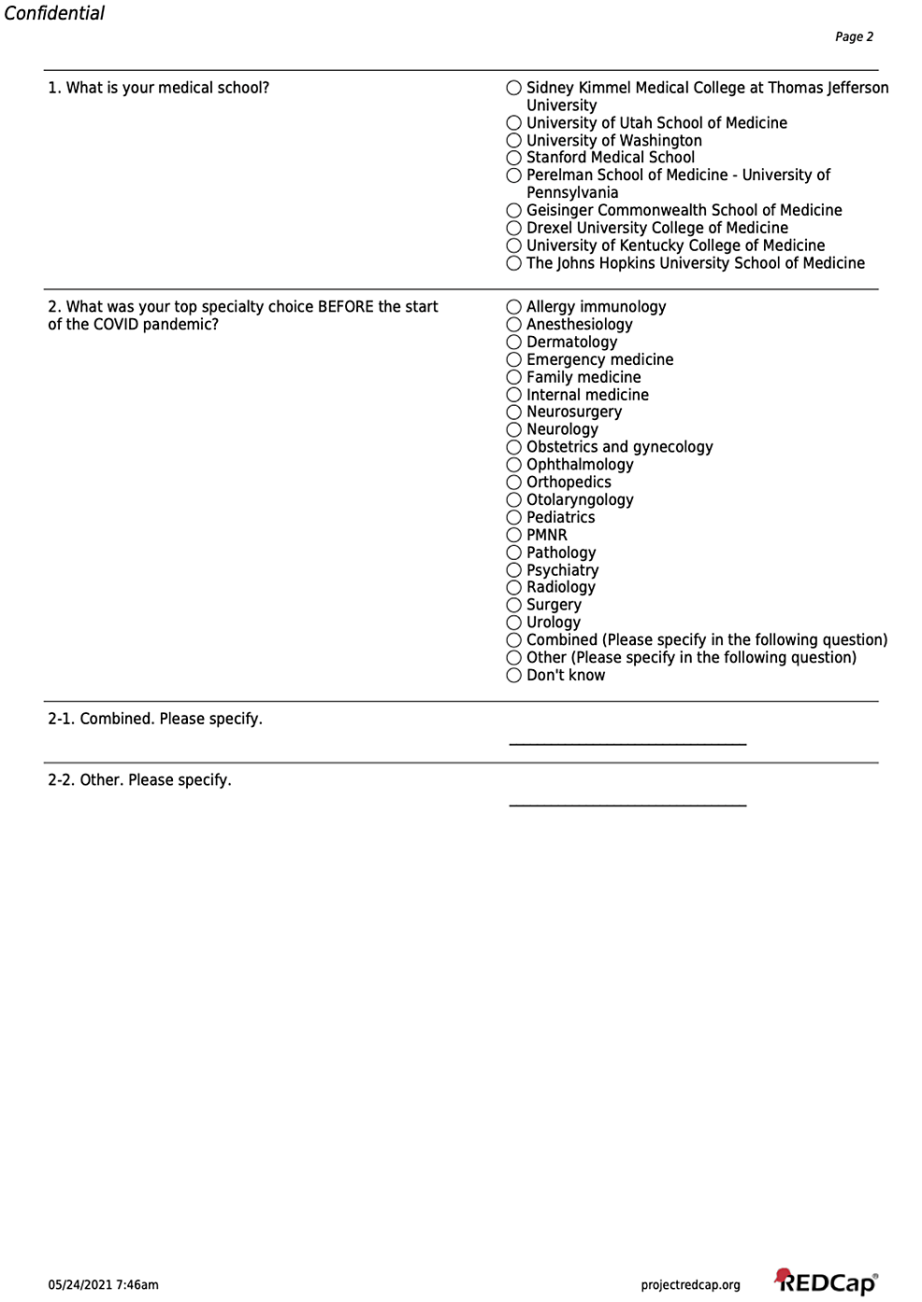
Survey Instrument Page 1

**Figure 2:**
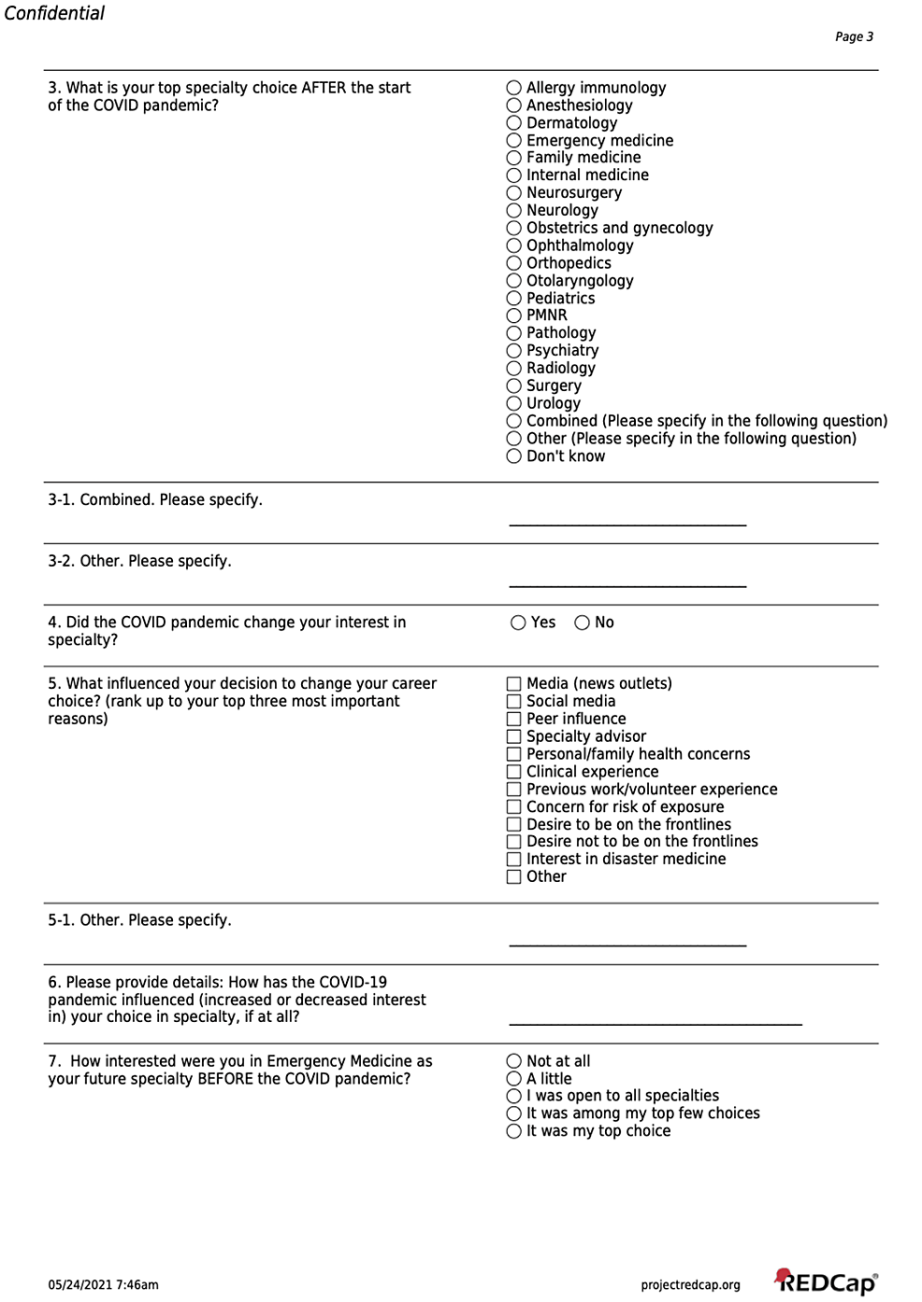
Survey Instrument Page 2

**Figure 3:**
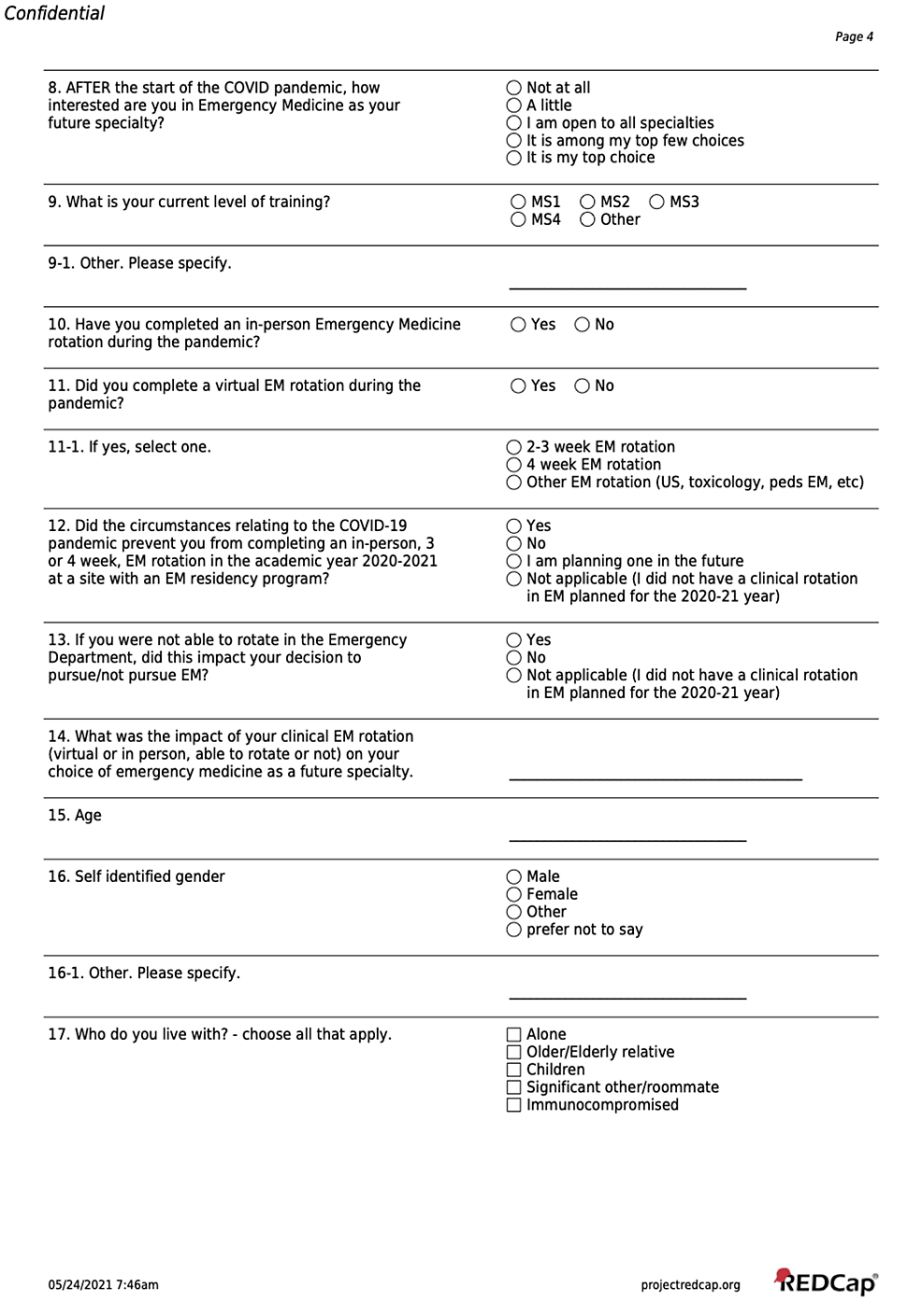
Survey Instrument Page 3

The survey instrument (Figures 1-3) was created by experienced medical educators at the constituent schools through a consensus-opinion method. Study authors met synchronously via online platform to develop a survey which aligned with the study aims and hypothesis. Survey questions were iteratively adjusted based on a small pilot sample of faculty and students from all schools, which were not included in the final survey and analysis. Informed consent of the study was provided in an email to solicited participants and online before the survey was started. The survey instrument asked students to indicate a primary specialty of interest both before and during the COVID-19 pandemic. Subsequent questions asked about the influence of the pandemic and related factors upon specialty choices, and among specialty choices related to Emergency Medicine.

Study setting and participant characteristics

All medical students, including those in combined programs (for example MD/PhD, MD/MPH, etc.), in all years of medical school, were eligible for participation. Student participation in the survey was voluntary, and consent was indicated by clicking on the survey link in the email. Participants were solicited for non-incentivized voluntary participation via email which was distributed within each medical school participating in the study. Survey links were distributed via email listservs at the constituent medical schools.

Study protocol

Anonymous and voluntary responses were collected using an online survey tool, REDCap 10.8.4. Students at each medical school were allowed three weeks to respond to the survey, with all students receiving weekly reminders to complete the survey. The survey was closed for students after the three-week window. The survey was available to each school in an asynchronous fashion, depending on school preferences and schedule limitations. The survey was sent out between December 2020 and January 2021. All students completed questions in the same sequence. Only surveys which were completed fully were included in our analysis.

Key outcome measures

Our key outcome measure was the degree of interest of medical students in a medical specialty, particularly emergency medicine, before the start of the COVID-19 pandemic and during the pandemic. Each student indicated a degree of interest.

Data analysis

We analyzed data using SAS v9.4 (Cary, NC, USA). Frequencies were calculated for response rates. Frequencies and relative changes were calculated for students who indicated a specialty preference for both before the pandemic began and for during the pandemic. Students’ levels of interest in their specialty were obtained using a five-point Likert scale and frequencies were calculated for level of interest both before and after the start of the COVID-19 pandemic. Chi-square analysis was performed to determine associations between career change and degree of interest in emergency medicine before and after the start of the COVID-19 pandemic as well as to determine the association between level of student training, and a change in specialty interest. A logistic regression analysis was performed to determine if age was associated with a change in specialty preference. Chi-square analyses were performed to determine whether an association existed between a change in specialty choice and each of the following variables: living alone, living with an older relative, living with children, living with a significant other, or living with an immunocompromised individual. The degree of interest in EM before the COVID-19 pandemic was compared to the degree of interest in EM during the COVID-19 using Bowker’s symmetry test and a Cohen Kappa value was calculated. A chi-square analysis was performed to determine the association between completion of an EM rotation, and degree of student interest in EM, both before and during the COVID-19 pandemic. Statistical significance levels were p< 0.05 for chi-squared analyses.

## Results

Students from nine medical schools from across the U.S. were surveyed. A total of 1431 of 6710 (21.3%) eligible students completed the survey (Table 1 shows individual school response rates).

**Table 1:** Surveyed schools and response rates The data has been represented as N (frequency column), % (Response rate per school, percent of Total Respondents columns)

Medical School	Location	Frequency	Response Rate per school	Percent Of Total Respondents
Sidney Kimmel Medical College at Thomas Jefferson University	Philadelphia, PA	261/1090	23.9%	18.2%
University of Utah School of Medicine	Salt Lake City, UT	272/490	55.5%	18.9%
University of Washington	Seattle, WA	171/1180	14.5%	12.0%
Stanford Medical School	Palo Alto, CA	35/488	7.2%	2.5%
Perelman School of Medicine - University of Pennsylvania	Philadelphia, PA	113/774	14.6%	7.9%
Geisinger Commonwealth School of Medicine	Scranton, PA	140/449	31.1%	9.8%
Drexel University College of Medicine	Philadelphia, PA	355/1041	34.1%	24.8%
University of Kentucky College of Medicine	Lexington, KY	26/718	3.6%	1.8%
The Johns Hopkins University School of Medicine	Baltimore, MD	58/480	12.1%	4.1%
Total		1431/6710	21.3%	100%

Among respondents, 786 (54.9%) identified their gender as female, and five (0.3%) identified as non-binary. A total of 1377 (96.2%) of respondents were between the ages of 22 and 32. A total of 1014 students provided their academic year in medical school which was evenly distributed (268 (26.4%) MS1, 246 (24.3%) MS2, 255 (25.2%) MS3, 231 (22.8%) MS4, 14 (1.38%) other (dual degree, research year, etc.)). Among students in the clinical phase of their education (MS3 and MS4), 275 (55%) had not completed an in-person EM clerkship at the time of the survey, and 26 (5.2%) had completed a virtual EM clerkship. Most virtual EM clerkships were two to three weeks long (n=15, 57.7%), with fewer four-week virtual clerkships (n=10, 38.5%). Forty-four (8.8%) students indicated that the COVID pandemic did impact their ability to complete an in-person EM clerkship at a site with an EM residency program. Of the students not able to complete an EM clerkship, 21 students agreed that the inability to complete the clerkship influenced their decision to pursue EM as a career choice.

The COVID pandemic was cited as a reason for a changed interest in specialty (could be increased or decreased interest) by 193 (13.5%) students surveyed. Among students whose specialty interest changed, the most frequently cited reason was the students’ clinical experience (102 (52.8%)) while the next most common reasons were a desire to be on the front lines (63 (32.6%)), and personal/family health concerns (46 (23.8%)). Table 2 shows the relative frequencies of responses to the rationale for specialty interest changes.

**Table 2:** Reasons for a change in specialty preference. (*up to 3 selections were possible and will not sum to 100%) The data has been represented as N (count), % (percent). Total responses were 1431.

Reason for change	Count	Percent of those changing specialty preference*
Clinical experience	102	52.9%
Desire to be on the frontlines	63	32.6%
Other	47	24.4%
Personal/family health concerns	46	23.8%
Previous work/volunteer experience	41	21.2%
Interest in disaster medicine	30	15.5%
Media (news outlets)	26	13.5%
Concern for risk of exposure	24	12.4%
Desire not to be on the frontlines	18	9.3%
Peer influence	17	8.8%
Social media	14	7.3%
Specialty advisor	10	5.2%

Narrative responses for choices not listed in the survey were also collected, with predominant themes including an inability to be exposed to EM clinical work, work-life balance concerns, medical school scheduling challenges during the pandemic, concerns about the nature of EM clinical work not related to the pandemic, and concerns about academic progress in light of the pandemic.

The overall rate of change of interest within each specialty from before the pandemic to after the pandemic is shown in Table 3.

**Table 3:** Frequency and Percentage of Change of Interest by specialty before and after the COVID-19 pandemic The data has been represented as N (count and count change), % (percent and percent change). Total number of students includes the entire study population (1431).

	Interest BEFORE the start of the COVID pandemic		Interest AFTER the start of the COVID pandemic		Change (AFTER - BEFORE)	
Specialty	Count	Percent	Count	Percent	Count Change	Percentage change
Allergy immunology	1	0.1	0	0.0	-1	-0.1
Anesthesiology	43	3.0	60	4.2	17	1.2
Dermatology	29	2.0	26	1.8	-3	-0.2
Emergency medicine	210	14.7	210	14.7	0	0.00
Family medicine	73	5.1	84	5.9	11	0.8
Internal medicine	219	15.3	232	16.2	13	0.9
Neurosurgery	22	1.5	25	1.8	3	0.3
Neurology	38	2.7	35	2.5	-3	-0.2
Obstetrics and gynecology	88	6.2	79	5.5	-9	-0.7
Ophthalmology	34	2.4	29	2.0	-5	-0.4
Orthopedics	87	6.1	73	5.1	-14	-1.0
Otolaryngology	32	2.2	19	1.3	-13	-0.9
Pediatrics	142	9.9	129	9.0	-13	-0.9
PMNR	16	1.1	12	0.8	-4	-0.3
Pathology	11	0.8	12	0.8	1	0.0
Psychiatry	56	3.9	61	4.3	5	0.4
Radiology	20	1.4	39	2.7	19	1.3
Surgery	134	9.4	136	9.5	2	0.1
Urology	21	1.5	19	1.3	-2	-0.2
Don’t Know	111	7.8	101	7.1	-10	-0.6
Internal Medicine-Pediatrics	16	1.1	21	1.5	5	0.4
Undecided	28	2.0	29	2.0	1	0.0

Chi-square testing determined there was a significant association between career change and degree of interest among students interested in emergency medicine as their future specialty before the COVID pandemic (𝞆2= 11.18, degrees of freedom (df) = 4, p=0.02) as well as after the COVID pandemic (𝞆2= 13.62, df = 4, p=0.01). Living with an immunocompromised individual had a significant association with a reduction of interest in specialty for emergency medicine (𝞆2 = 4.25, df = 1, p-value=0.04).

Across all specialties, Chi-square analysis showed that the level of training of the student did not affect the change of interest in specialty (𝞆2= 0.82, df = 1, p=0.37). Logistic regression analysis showed that age was not a significant covariate in relation to whether the COVID pandemic changed student's interest in specialty (Wald 𝞆2 = 0.72, df=1, p=0.40). Whether a student lived alone, with an older relative, children, or significant other/roommates had no significant association with a change of interest in specialty (p> 0.05 for all). Table 4 shows the shift in interest with respect to EM as the COVID-19 pandemic took place.

**Table 4:** Relative shifts in student interest in EM before and after the start of the COVID-19 pandemic The data has been represented as N for all columns and rows.

Degree of interest in EM BEFORE the COVID pandemic	Degree of interest in EM AFTER the start of the COVID pandemic					
	Not at all	A little	I am open to all specialties	It is among my top few choices	It is my top choice	Total
Not at all	417	29	1	5	1	453
​​A little	68	259	17	16	5	365
I was open to all specialties	22	39	108	26	4	199
It was among my top few choices	11	29	17	172	44	273
It was my top choice	3	3	1	21	112	140
Total	521	359	144	240	166	1430
Missing = 1						

With respect to students’ specific interest in emergency medicine, analysis showed that 214 students (15%) became less interested in EM whereas 148 students (10.4%) became more interested in EM (Table 5).

**Table 5:** Favorability towards EM before and after the start of the COVID-19 pandemic The data has been represented as N (Total Count), % (Percent)

Interest change	Total Count	Percent
Became less favorable to EM	214	15.0
Stayed the same	1068	74.6
Became more favorable to EM	148	10.4

Overall interest very slightly declined (-0.08% for the entire data set and -0.31% among those who changed their rating of EM). Bowker’s symmetry showed at least one pair of cells was not symmetric (𝞆2=62.82, df = 10, p<.01) affirming this overall decrease in interest in EM. Cohen's Kappa was 0.77 (95% CI 0.75-0.79) for the overall data set, indicating there was substantial agreement between how interested students are in emergency medicine as their future specialty before and after the COVID pandemic (Table 5).

Chi-square testing showed there was a significant association between EM rotation completion and how interested students are in emergency medicine as their future specialty before the COVID pandemic (𝞆2= 30.04, df = 4, p < 0.01) and after the COVID pandemic (𝞆2 = 55.40, df =4, p < 0.01).

Among students whose specialty interest was changed by the COVID pandemic, 34 (41.5%) became less favorable to EM, 28 (34.2%) stayed the same, and 20 (24.4%) students became more favorable to EM. On the other hand, among students whose interest in specialty was not changed by the COVID pandemic, 97 (23.3%) became less favorable to EM, 271 (65%) stayed the same, and 49 (11.8%) students became more favorable to EM. The former group became much less favorable to EM than the latter group on average (-0.40% vs. -0.18% for the entire data set and -0.61% vs. -0.51% among those who changed rating of EM).

Among students who had completed an in-person EM rotation during the pandemic, 64 (28.6%) of students became less favorable to EM, 123 (54.9%) stayed the same, and 137 (6.5%) became more favorable to EM, respectively. On the other hand, among students who had not completed an in-person EM rotation during the pandemic, 67 (24.4%) of students became less favorable to EM, 176 (64.0%) stayed the same, and 32 (11.6%) became more favorable to EM. Moreover, both groups had negative average changes in interest in EM as a future specialty between before and after COVID pandemic. In addition, for both groups, Bowker’s symmetry test showed at least one pair of off-diagonal cells was not symmetric (𝞆2= 56.01, df = 10, p-value <.001), which affirmed a significant change.

## Discussion

As the full impact of COVID-19 on specialty interest continues to come to light, early work suggests that for a large proportion of students, the pandemic has played a key role in their choice of career, which was supported by our findings [6,12]. The 2023 National Resident Matching Program (NRMP) results for Emergency Medicine showed a significant decline in applications and matched positions [12]. This recent NRMP data, paired with prior research and our work here, supports the concept that medical student specialty choice is influenced by a vast array of personal and professional components including exposure via clinical rotations, perceived professional values, expectations of the practice environment, and lifestyle considerations [3,13]. A recent meta-analysis of factors influencing specialty choice among medical students was written before the pandemic and therefore was unable to analyze or incorporate the effects of the pandemic on specialty choice [14]. Our study therefore adds to this foundation of knowledge.

While we did not see any large changes in student specialty selection secondary to the COVID-19 pandemic, our study did demonstrate several interesting shifts in interest. For instance, there was an increase of interest in radiology and internal medicine, but a decrease of interest in orthopedics and otolaryngology as career choices. One could hypothesize that students surveyed have developed increased interest in fields that provided safer work environments with less exposure to COVID-19, such as radiology. Just like practicing physicians, students have had to consider how their work environment and its associated risks might impact their family. In fact, 45 students cited changing their career choice because of either personal or family health concerns [15]. On the other hand, of those who reported changing their career plans because of COVID-19, 4.5% of them did so because they wanted to be working on the frontlines. This shift and associated rationale may help explain the increased interest of students in internal medicine who were seen to be working on the frontlines treating patients during the pandemic.

It is very likely that exposure to clinical experiences played a role in these shifts. Professional identity formation is a key process that helps medical students choose their career path. Recent literature has described the potential effects of the COVID-19 pandemic on students’ abilities to develop their professional identity [16] and suggests that underdevelopment of professional identity may occur as a result of the suspension of clinical rotations, which may also lead to career dissatisfaction and burnout [17]. Students reported that lack of exposure was the most common reason for career change as it related to COVID-19 in our survey, and this has been cited as a common concern by students in other recent works [6]. In our study, this was particularly apparent in emergency medicine interest as a lack of clinical exposure was associated with a negative shift in interest. In addition, given that elective orthopedic and otolaryngology surgical cases were postponed in many parts of the country, it is possible that students did not have enough exposure to these specialties to feel comfortable choosing them as career options. In fact, educators in otolaryngology identified this potential problem early in the course of the pandemic and published a call to action for addressing this educational gap [18].

Given these identified trends in other specialties, one might have expected a shift in those interested in emergency medicine secondary to the pandemic. We might have expected a decrease in interest in EM with preference for specialties with less risk and exposure. At the same time, others may have an increased interest given the role providers play in caring for patients on the frontlines. Our study in fact found a slight overall decrease in EM interest amongst students, but we could not account for simultaneous opposite shifts in interest which may have occurred. It is unclear why there was a predominant decrease in interest as all of the influences we examined, with exception of living with an immunocompromised individual, did not have a significant impact on decisions. Similarly, we found that medical student (MS) level did not have a significant impact on this group.

Prior research has suggested that students choose an emergency medicine career for a variety of reasons including positive perceptions of the work. Those students who chose a career in EM perceived the work of the EM physician as “interesting, exciting, wide-ranging” and they aligned those characteristics with their own values [19]. Although this decrease in EM interest was slight, the long-term impact of the COVID pandemic on student specialty interest remains to be seen and may be difficult to discern given confounding factors. Qualitative analysis of narrative data will reveal a better understanding of factors that impacted student decisions with regard to specialty choice. Unfortunately, future studies may be confounded by a recent report highlighting a future surplus of emergency medicine physicians. It is reasonable to predict a further decrease in EM interest based on predicted job market trends in emergency medicine [20].

Limitations

This was a survey-based study inclusive of nine different medical schools. Although the schools included in the study represent diverse populations and training environments, this study is prone to selection bias and may not be representative of all medical institutions. Our study is limited by an overall low response rate and a variable response rate between schools (7.2%-55.5%) which also could lead to bias. We were not able to capture differences among medical school class levels for all specialties, only for EM-bound students. Although all sites sent emails through listservs it is possible that emails bounced back or went to junk mail, furthering potential bias. As with many survey studies, we are limited by the potential for recall bias from participants. Also, it is plausible that respondents may have completed the survey more than once and were not identified in our analyses due to the confidentiality processes included in our study. Finally, this survey-based study provides important insights as to the impact of the pandemic on student specialty choices but is limited in providing understanding as to what may have led to students’ decisions. Future qualitative analyses of narrative data in our study will be necessary to further confirm and inform our findings. This would better inform advisors on how to prepare students to identify and pursue their future specialty even when we are not in the midst of a pandemic.

## Conclusions

The impact of COVID-19 on medical students’ career choice is a complex issue that involves both personal and professional factors. It does appear that there is a trend towards less interest in the field of EM during the COVID-19 pandemic. However, the survey data also indicate that effects on student decisions are fluid, with some expressing an increased interest in EM despite COVID. It is anticipated that over the next few years, we will truly see the effects that a global pandemic has on students’ career choices within medicine and specifically within EM.
